# Editorial: Molecular Strategies Aimed to Boost NK Cell-Based Immunotherapy of Cancer

**DOI:** 10.3389/fimmu.2020.01132

**Published:** 2020-06-16

**Authors:** Loredana Cifaldi, James Di Santo, Daniel Olive

**Affiliations:** ^1^Academic Department of Pediatrics (DPUO), Ospedale Pediatrico Bambino Gesù, IRCCS, Rome, Italy; ^2^Innate Immunity Unit, Institut Pasteur, Paris, France; ^3^Institut National de la Santé et de la Recherche Médicale (INSERM) U1223, Paris, France; ^4^Cancer Research Center of Marseille, INSERM U1068, CNRS U7258, Aix-Marseille University, Institut Paoli-Calmettes, Marseille, France

**Keywords:** NK cells, NK cell-based immunotherapy, cancer immunotherapy, immune checkpoint inhibitors, adoptive transfer of NK and CAR-NK cells, ligands for NK cell-activating receptors

Natural Killer (NK) cells represent the first line of defense against aberrant cells, playing a crucial role in counteracting tumor development. Although NK cells are able to circulate in many tissues and rapidly recognize and eliminate tumor cells, a combination of deregulated molecular networks, occurring in tumor microenvironment (TME), allows the immune escape of tumor cells from NK cell-mediated surveillance, thus contributing to tumor progression. The expression of immune checkpoint molecules contributes to the functional exhaustion of NK cells. To overcome the NK cell limitations in the fight against tumor cells, several molecular strategies have been recently adopted at a preclinical and clinical level. Therefore, the use of monoclonal antibodies (mAbs) that neutralize the immune checkpoint molecules and the adoptive transfer of *ex vivo* expanded and activated NK cells or chimeric antigen receptor (CAR)-modified NK cells represent the main NK cell-based immunotherapeutic approaches currently adopted to treat tumors. Of note, NK cells, unlike T cells, do not cause graft-versus-host disease (GvHD) allowing a safe and successful NK cell adoptive transfer in an allogenic setting. Furthermore, although the use of CAR-T cells has shown potent antitumor efficacy, their application is restricted to an autologous setting. Therefore, CAR-NK cells constitute an off-the-shelf product with enormous immunotherapeutic potential.

All of these issues have been extensively described in the reviews collected in this Research Topic, also raising several questions about approaches not yet fully investigated.

Sun and Sun claimed that blocking with checkpoint inhibitors can restore functional exhaustion of NK cells and may complement the limitation of T cell-based immunotherapy. The authors described preclinical studies and clinical application of neutralizing mAbs targeting NKG2A, KIRs TIGIT, CD96, LAG-3, and TIM-3 NK cell-inhibitory receptors.

Minetto et al. focused on the expression of HLA-I on tumor cells and how it can influence the anti-tumor NK cell-mediated functions. These authors described recent NK cell-mediated anti-tumor clinical approaches based on the use of mAbs recognizing NK cell-inhibitory receptors and immune checkpoint inhibitors, alone or in combination with other compounds.

Bi and Tian described the benefits mediated by immune checkpoint inhibitors on NK cells. The authors argued that PD-1^+^ NK cells, displaying a stronger potential than PD-1^−^ NK cells, can be functionally compromised by PD-L1 which is highly expressed in the TME. Therefore, PD-1/PD-L1 blockade can restore PD-1^+^ NK cell functions. In addition, the authors proposed the therapeutic targeting of other molecules involved in functional suppression of NK cells, such as E3 ubiquitin ligase Cbl-b, IL-1R8, the negative regulator of IL-15 CIS, and A2A adenosine receptor.

In addition to these approaches, Zhang et al. proposed other strategies to overcome the NK cell suppression in TME, such as the clinical use of TGF-β neutralizing mAb Fresolimumab and TGFβR1 inhibitor Galunisertib in solid tumors. The authors contemplated the use of NK cells knocked down for *TGFBR2* or *SMAD3*, or the use of CAR-modified NK cells containing TGF-β type II receptor. In addition, they envisaged the use of CMV-induced adaptive memory NK cells and CAR NKG2C^+^ CD57^+^ adaptive NK cells for clinical implication.

Vacca et al. focused on different strategies adopted for NK cell-based immunotherapy. In addition to the use of cytokines, mAbs and checkpoint inhibitors, all factors that increase the efficacy of the adoptive transfer of NK cells and CAR-NK cells, the authors underlined the efficacy of αβT cell- and B cell-depleted haploidentical hematopoietic stem cells (HSC) transplantation in which the infusion of donor NK cells and γδT cells, together with HSC, resulted in reduced leukemia relapses and infections.

Melaiu et al. focused mainly on solid tumors and described the immune evasion mechanisms occurring in TME leading to the NK cell exhaustion, together with the preclinical and clinical NK cell-based immunotherapeutic studies. They described the susceptibility of cancer stem cells (CSCs) to NK cell-mediated killing as evaluated in preclinical models. Moreover, they reported studies on NK cell distribution, phenotype, and function in several solid tumor tissues.

Molfetta et al. described other immune evasion strategies, such as the downregulation and cleavage of ligands for NK cell-activating receptors through transcriptional, post-transcriptional and post-translational mechanisms. The authors argued that chemotherapy drugs known to inhibit the ubiquitination and SUMOylation, which are mechanisms involved in the negative regulation of ligands for NK cell-activating receptors, may be exploited for their immunomodulatory effects, thus suggesting their clinical use in combination with conventional therapies.

Sayitoglu et al. in a research article reported new data of genetically modified (GM)-NK-92 cells activity in sarcoma. The authors explored the profile of ligands for NK cell-activating receptors in sarcoma primary cells and underlined the importance of PCNA, CD112, and CD115 expression for NK cell-mediated recognition. They developed a screening platform to assess the efficiency of 14 different activating receptors by generating GM-NK-92 cells over-expressing each individual receptor. The authors show that DNAM-1 and NKG2D GM-NK-92 cells elicited an increase NK cell-degranulation and cytotoxicity against primary sarcoma cells. Moreover, DNAM-1 GM-NK-92 degranulated efficiently also in response to other solid tumor cell lines.

Heinze et al. in a research article described the NK cell anti-tumor efficacy against neuroblastoma (NB) cells by comparing cytokine-induced killer (CIK) with *ex vivo* expanded NK cells, activated with IL-2, IL-15, and IL-21. The authors compared also two different protocols of NK cell isolation and showed that CD3/CD19-depleted PBMCs NK cells expanded higher that CD56-enriched cells. NK cells were assayed in cytotoxic activity against NB spheroidal culture. In addition, IL-21, in combination with IL-15, enhanced NK cell proliferation thus suggesting that the use of IL-15+IL-21 expanded NK cells from CD3/CD19-depleted PBMCs may be promising for a new NK cell-based immunotherapy.

Müller et al. in a research article optimized the protocol leading to the generation of PBMC-derived CD19-CAR-NK cells. These authors compared lentiviral and alpharetroviral, in combination with two different transduction enhancers such as Retronectin and Vectofusin-1. They demonstrated that the transduction of primary NK cells with RD114-TR pseudotyped retroviral vectors, in combination with Vectofusin-1, generated CD19-CAR-NK cells with a highly efficient cytotoxicity against CD19-expressing lymphoblastic cells.

Last but by no means least, we would like to dedicate this series of NK cell-based immunotherapy papers to the memory of Professor Vito Pistoia (Pediatric Hospital Bambino Gesù, Rome) who tragically passed away in 2018. This collection is a fitting tribute to his creative scientific contributions and his unwavering dedication to the field of Cancer Immunity and Immunotherapy.

## Author Contributions

All authors listed have made a substantial, direct and intellectual contribution to the work, and approved it for publication.

## Conflict of Interest

DO is the co-founder and share holder of company Imcheck Therapeutics. The remaining authors declare that the research was conducted in the absence of any commercial or financial relationships that could be construed as a potential conflict of interest.

